# Decisional Dimensions in Expert Witness Testimony **–** A Structural Analysis

**DOI:** 10.3389/fpsyg.2018.02073

**Published:** 2018-10-31

**Authors:** Alex Biedermann, Kyriakos N. Kotsoglou

**Affiliations:** ^1^School of Criminal Justice, Faculty of Law, Criminal Justice and Public Administration, University of Lausanne, Lausanne, Switzerland; ^2^Litigation Law Unit, University of Adelaide Law School, Adelaide, SA, Australia; ^3^School of Law, Liverpool Hope University, Liverpool, United Kingdom

**Keywords:** expert evidence, legal process, decision analysis, normative approach, decision-making prerogative, expert witness fallacy

## Abstract

The relationship between forensic science and legal adjudication is intricate mainly because the need to inform fact-finders on issues going beyond the layman’s knowledge poses challenges both on empirical and normative dimensions, in particular with regards to the specific role and duties of the different participants in the legal process. While rationality is widely upheld as one of the aspirations of the legal process across many modern jurisdictions, a pending question is how to remedy the uneasy relationship between general propositions (and knowledge claims) conditioning expert witness testimony, and individualized decisions taken by fact-finders. The focus has hitherto been put on the utilization of model-based and formal methods of reasoning while, regrettably, the concepts of judgment and decision-making have not received equal attention. A first aspiration of our paper will thus be to further clarify the nature of this systemic relationship in the particular area of the legal process involving scientific experts, by conducting a critical transversal analysis of current empirical, normative and doctrinal understandings of expert witness testimony. As a second aim, we will use this insight to argue in favor of the view that structural features of expert witness testimony are embedded in a decision-making process, and that the understanding of this decisional dimension is important for clarifying the respective roles of expert witnesses and fact-finders, and for favoring their mutual understanding thereof. To substantiate this perspective, and attest to its growing recognition as a frontier understanding, we will provide real-world examples from forensic science reporting practice and policy documents of professional bodies.

“[Y]our degree of belief does not, by itself, dictate what you should *say* or *do* (…) A rational decision about what to do requires more than the evidence you have” (Sober, 2008, at p. 7)

## Introduction

In law, as much as in other disciplines, the topics of judgment and decision making (JDM) under uncertainty have both a long-standing and lively debated history, on all common levels of consideration, normative^1^, descriptive and prescriptive. Qualitative verbal decision criteria, such as ‘beyond reasonable doubt’ (BRD), are typical examples that lend themselves for study under these distinct perspectives. Most textbooks in the field present standard legal decision criteria, but the competing interpretations of the nature and logical structure of such terms divides practitioners and scholars since decades. Normativists – to name one of the groups of discussants– have analyzed and expressed legal standards of decision in terms of formal frameworks, such as (Bayesian) decision theory, since the 1960s (i.e., Kaplan, 1968). Their research led to interesting analytical results, some of which confirm the meaningfulness of conventional decision standards, such as the >50% probability requirement for finding civil liability (e.g., Friedman, 1997; Kaye, 1999). In such normative frameworks, minimal probability thresholds required to justify particular decisions are tightly bound to preferences among decision consequences through so-called consistency relationships (e.g., Buchak, 2016). Formally, these relationships amount to principles such as minimizing (or maximizing) expected loss (or utilities). Substantial amount of research (for a review see, e.g., Connolly, 1987) has been devoted to the empirical investigation of the extent to which people’s actual thinking and deciding in legal applications aligns to such consistency relationships between, on the one hand, beliefs about competing versions of the event of interest (i.e., hypotheses or propositions), expressed in terms of probabilities, and on the other hand preferences among decision outcomes^2^, expressed in terms of utilities or losses. Such empirical work found that there is a considerable mismatch between the decisions that individuals were willing to make, that is decision behavior, and the decisions that would be optimal according to the normative account.

Further investigation of these results seems to have come to halt because of the exhausted perspectives that they represent. Normativists, for example, argue that precepts following from logical considerations simply are not and cannot be invalidated in principle by any mismatch with practically observable decision behavior that there may be. According to this view, the poor mapping of formal theories on legal adjudication does not represent a failure, because description and explanation is not one of the aspirations of these theories. Empiricists, in turn, consider aspects of normative perspectives pointless because of the absence of legal requirements in the first place that would ask participants in the legal process to conform to aspects such as the maximization or minimization of an expectation of any quantity of interest, such as utility or loss (e.g., Allen, 2003). This suggests that there is an impasse between the reality faced by legal practitioners and the many conceptual accounts offered by decade-long legal research and scholarship in formal approaches to inference and proof, including empirical studies by experimental psychologists. Hence, contributing to a collection in the area of judgment and decision making in this journal of applied psychology poses not only a high burden of providing original discussion. It also requires a clear statement of the scope of enquiry within the broad perspectives of descriptive, normative and prescriptive research.

We address this challenge by focusing our attention not to the process of legal adjudication as a whole, in particular ultimate issues to which most of the abovementioned decision-theoretic research relates, but to the intersection between forensic science and legal adjudication, in particular the form, content and elicitation of forensic expert conclusions. The reason for this is that, first, while debates over the appropriate approach to the various dimensions in which legal adjudication seeks optimization^3^ seem stalled, there are local instances of the legal process, such as the use of specialized (forensic science) evidence, that represent unresolved conceptual difficulties in practical proceedings. Some of these difficulties are peculiar to the intersection between science and the law, such as the deferential versus educational approaches to deal with specialized knowledge in the process (e.g., Miller and Allen, 1993). A second reason is that devising a coherent approach to such challenges is an important preliminary to sound decisions at higher levels in the process, of which forensic expertise may be an integral part. By forensic evidence we mean, throughout this paper, both physical/chemical and digital *non-replicable* items of evidence, usable to help recipients of expert information discriminate between competing propositions of interest, or help reduce the pool of potential persons/objects at the origin of a particular trace or item seized in relation to an event of legal interest (criminal, civil, or administrative). Examples for particular types of forensic traces are mentioned in Section “Decision-Structures for Specific Evidence in Forensic Science.”

In essence, our analysis will come down to, and articulates what we will call ‘decision-structures.’ We show that decision-structures, although normative in nature, conceptualize and ascribe content to existing adjudicative practice, for they capture the requirement of ‘specific evidence.’ Central to this argument is that the proposed decisional perspective is not an end in itself, but only a necessary preliminary to understanding the reason for and justification of counter-current positions, such as the call to abandon some traditional expert reporting formats; especially categorical conclusions that usurp the epistemic rights of fact-finders. This result will call into question the extent and scope of some of the current and most longstanding forensic science reporting schemes. To redirect such forensic testimony on its proper track, recipients of expert information need to assume a more active role in the processing of scientific evidence, by insisting on their role as ultimate arbiters of probative value in criminal trials, in particular by explicating their exclusive epistemic duty to reach contextually structured decisions.

Methodologically, we will rely on the view according to which the logical and balanced assessment of scientific evidence is a central part of forensic expert testimony. Though, traditionally, this is said to involve probability as a measure of uncertainty (e.g., Aitken et al., 2010), we will adopt a broader perspective here and consider forensic expert testimony as an instance of a normatively structured decision-making process under uncertainty. Thus, we regard forensic expert testimony not as an abstract and isolated object of inquiry but blend it with considerations from actual forensic practice (e.g., policy documents and practitioner guidelines). This will also prompt us to assess the ways in which this perspective may contribute to the improvement of frontier understandings about the processing of scientific evidence in legal adjudication.

Our paper is structured as follows. Section “Transversal Overview of Current Empirical, Normative and Doctrinal Understandings of Expert Witness Testimony” critically reviews current perspectives on expert witness testimony. Using practical examples, we will expose areas of interaction between forensic science and the law where conflicting views about the form and the content of expert testimony continue to pose challenges for the legal resolution of disputes. Based on this initial diagnosis we will argue, in Section “Decisional Dimensions of Forensic Expert Testimony,” that considering expert testimony not merely as an inference problem, but analyzed as a contribution to a process of decision, dissolves key aspects of current controversies without breach with either logical considerations or procedural principles. Discussion and conclusions are presented in the last section.

## Transversal Overview of Current Empirical, Normative and Doctrinal Understandings of Expert Witness Testimony

### Key Controversies Over Selected Aspects of Forensic Expert Testimony

Traditionally, forensic science is regarded as a collection of applied scientific methods and techniques for the purpose of assisting the judiciary in specialized matters where it lacks relevant knowledge and expertise. While science and technology are subject to continuous change and development, conceptual questions gravitating around the quantification and weighing of scientific findings tend to concentrate on a singular, well settled perspective. Evett (2009, p. 159, emphasis as in original) has expressed this perspective as follows: “the single most important advance has nothing to do with technology [...]. It tells us the most important lesson for the logic of evaluative forensic science: *consider the probability of the evidence, given the proposition.*” How could such a simple sentence – at least at first sight – be considered the most important lesson for evaluative forensic science? A main reason is that it clearly delineates the area of competence of the expert, as noted by Margot: “[w]hether these results [are] observed if one proposition for the event is true rather than another proposition is the central relevant matter on which the forensic scientist may comment” (Margot, 2011, p. 796). This focus is fundamentally different from that of fact-finders who concentrate on propositions, given the evidence (Robertson et al., 2016). Yet these different logical conditionings, in particular evidence *given* target propositions (and the reverse), trouble both scientists and recipients of expert information since decades (Thompson and Schumann, 1987): it is the archetype forensic science and legal adjudication example for a normatively sound principle that is practically poorly understood. Many past and recent initiatives, including efforts by renowned scientific societies (e.g., Aitken et al., 2010), focus on explaining and exemplifying these principles through guidelines, recommendations and primer documents (e.g., The Council of the Inns of Court [COIC] and The Royal Statistical Society [RSS], 2017).

Often, however, the above state-of-the-art occupies only a side-arena of broader debates over forensic conclusion formats, with different discussants pulling the rope in different directions. Proponents in forensic fields that pursue the idea of identification (also sometimes called ‘individualization’), provide typical examples for this. Identification, in the present context, is widely understood as the reduction of a pool of potential sources (of a crime stain, mark or trace) to one and only one single candidate (i.e., a person, object or tool). Examples of traces are biological stains (e.g., blood, saliva, etc.), marks on fired bullets, bite-marks, handwriting/signatures etc. and examples of conclusions are ‘this DNA comes from *this* person,’ or ‘*this* mark comes from this tool/person’ etc. The unscientific character of such categorical conclusions (i.e., certainty assertions) has been prominently exposed by Stoney (1991) in his landmark paper “What made us ever think we could individualize using statistics?”, but remains widely unrecognized. Not only are identification/individualization conclusions by scientists logically untenable, it has also been shown empirically that forensic examiners, in many instances, cannot make such determinations reliably, or at least exhibit a potential of error. This has encouraged calls to initiate a paradigm shift (e.g., Saks and Koehler, 2005), but the effect merely was to keep the topic on the agenda, leaving fundamental changes by practitioners pending, even in the light of subsequent, critical reports by the National Research Council [NAS] (2009) and, more recently, the President’s Council of Advisors on Science and Technology [PCAST] (2016).

This divide over forensic reporting formats also surfaces on institutional levels, revealing the profound gaps between legal and scientific proponents. Most recently, the Department of Justice released a document entitled “Approved uniform language for testimony and reports for the forensic latent discipline” (U.S. Department of Justice [DOJ], 2018) which, contrary to the above considerations, upholds bold statements such as “identification” and “exclusion”.^4,5^ This does not fit well with the release, in 2016, by the Office of the Attorney General (U.S. Department of Justice), of a memorandum to advise *against* categorical conclusions (certainty conclusions of the type mentioned above): “Department forensic laboratories will review their policies and procedures to ensure that forensic examiners are not using the expressions “reasonable scientific certainty” or “reasonable [forensic discipline] certainty” in their reports or testimony. Department prosecutors will abstain from use of these expressions when presenting forensic reports or questioning forensic experts in court unless required by a judge or applicable law” (Office of the Attorney General, U. S. Department of Justice [OAG], 2016). While this seems to be a clear message, the position of scientists remains far from uniform. For example, in a position statement regarding the OAG memorandum, some practicing DNA scientists re-asserted their adherence to categorical reporting formats (i.e., identifications), called ‘source attribution determinations’ (e.g., Moretti and Budowle, 2017). Unstated, however, remains the fact that this statement is based on particular assumptions that, in operational casework, are highly debatable (e.g., the omission of the potential of error), thus making the position both peculiar and difficult to defend. More generally, positions of this kind represent only one side of the extremes that characterize the concurrent streams of development in forensic science. Suffice to notice, as a counter example, that Cole (2014, p. 144) concluded, in a meticulous review of forensic (fingerprint) analysis practice, that “forensic identification will have difficulty moving forward until ‘individualization’ is really dead.” Such stark language also continues to emanate from most recent discussions. A concise illustration for this is given by Evett who, at a NIST colloquium in 2017, has been quoted as saying “The identification paradigm is going to die, because as scientists we realize there’s no basis for it” (Champod and Evett, 2017).

Despite these fundamentally opposed views on forensic expert conclusions, there is one common thread to which all discussants appear to subscribe: the idea of contributing to sound decision-making. Yet, strangely, current discussions in both practice and literature almost exclusively focus on questions about the nature, foundations and internal consistency of expert witness testimony, leaving aside the crucial question on how testimony interfaces with decision-making in the wider, albeit structured and detailed context of legal adjudication. We will critically expose this interface in further detail in Section “Decisional Dimensions of Forensic Expert Testimony,” introducing the notions of decisional dimension and forensic decision structures. It is first necessary, however, to introduce elements from law (see Section “Law of Evidence, Complexity and Decision-Making Prerogatives”), in particular evidence law, and considerations of how legal orders deal with science, as exemplified by landmark decisions such as *Daubert*, followed by its subsequent discussion by legal commentators (see Section “Law and Philosophy of Science”). These preliminaries aim at providing the wider scene wherein which the decisional dimension of expert witness testimony, presented in Section “Decisional Dimensions of Forensic Expert Testimony,” is to be understood.

### Law of Evidence, Complexity and Decision-Making Prerogatives

As a preliminary, it is important to recognize that specialized forms of evidence, such as forensic science evidence, are merely instances of the broader challenge of evidence processing. Notwithstanding, the intersection between forensic science and legal (especially criminal) adjudication is often considered a prime example for illustrating the ‘problem’ of specialized knowledge, generally termed expert evidence (or, expert witness testimony) throughout this paper. It is commonly understood that the need to inform fact-finders on issues going beyond the layman’s knowledge (see Section “Key Controversies Over Selected Aspects of Forensic Expert Testimony”) poses challenges both on empirical and normative dimensions, in particular with regard to the specific role and epistemic duties and rights of the different participants in the legal process.

The very possibility of accurate and efficient legal operations hinges on the ability of fact-finders to recognize particular acts and circumstances as instantiations of abstract legal concepts. It is widely recognized that this capacity has reached new limits in today’s technology-driven modern world, with its wide range of socioeconomic activities. What is more, it is questionable whether laypeople can appropriately comprehend evidential items or phenomena and assess their informative contents with respect to the contested facts of the case, when this requires trained expert sensory capacities and specialized knowledge. The technological advances of our age raise thus pressing questions of competence: who should have the *decision-making prerogative* regarding selected conclusions (e.g., regarding the origin of a particular mark or stain, item of handwriting, etc.) when dealing with items of evidence that require knowledge fact-finders do not usually possess? This central issue will be addressed in later Sections of this paper.

Interestingly, from a historic point of view, this is not the first time that the increasing complexity of society and compartmentalization of human knowledge place additional strain on legal systems and the “good old way,” according to which expert witnesses act simply as “helpers of the court” (Thayer, 1892, p. 665). Legal history gives relevant insight into the dynamics of the law of evidence (Golan, 1999). During the Middle Ages, jurors in predominantly agricultural societies – whose level of sophistication and technological advance was not radically different from that of ancient communities – were drawn from the immediate surroundings of the accused (Langbein, 1996, p. 1170). The rationale underpinning this adjudicative structure was the assumption that the jurors would either be familiar with the allegation at play or they would be able to investigate on their own. As small communities gave their place to increasingly larger ones, the institution of self-informing jurors underwent fundamental changes, for the law covered gradually broader areas of social life. There was simply too much information to navigate and it was now passive fact-finders, ignorant of the contested facts and essentially dependent on witness testimony, who decided cases. These laypeople with no direct knowledge of the facts were, obviously, in need of judicial instructions regulating the routines for gathering and assessing evidence, i.e., information, in order to render a verdict. According to John Henry Wigmore, the law of evidence grew, at this very moment, as procedural necessity and doctrinal reality. It was, in other words, the dawning of the instructional trial propelled by the need to acquire (specialized) information that molded the law of evidence (Wigmore, 1908, p. 692).

The complexity of legal proof seems to swing the pendulum back in the direction of active rather than passive fact-finders, suggesting that they are wearing this time the hat of ‘expert witnesses.’ For the democratically legitimized and from the legal order authorized professional judges or jury cannot be the ones, so a general claim in forensic science, who make so-called identification decisions (as defined in Section “Key Controversies Over Selected Aspects of Forensic Expert Testimony”). According to the deference model, fact-finders will have – on the pain of irrationality – to delegate some of their cognitive monopoly to experts, at least every time the contested facts feature questions encroaching beyond the boundaries of what is commonly known.

On a practical account, however, the notions of decision-making prerogatives and deference lead to a critical impasse. On the one hand, the procedural necessity of filling abstract legal terms with valid (and reliable) empirical content highlights the systemic relationship between legal adjudication, especially criminal adjudication, and (forensic) science. For one of the central tenets of modern legal orders, the Rationalist Tradition, is the requirement that all decisions, which affect the interests of individuals by resolving disputed questions of fact, are justifiable (Twining, 1982). A decision-making process in which the fact-finder does *not* properly understand the nature (e.g., statistical) and empirical content of evidence would be arbitrary and have deleterious effects for the public confidence in the integrity and accuracy of the legal system. Lord Steyn’s dictum that “[c]ourts of law can only act on the best scientific understanding of the day”^6^ entails the admission that fact-finding can be as good as modern science allows it to be. On the other hand, the relationship between the two symbiotic partners is characterized by friction and antagonism. Forensic scientists take the legal axiom ‘iudex non calculat’ quite literally and deplore that traditional fact-finders (judges and jurors) struggle with the proper understanding of scientific methods, and science in general. At the same time, the state of forensic science causes itself, in regular intervals, scorn, and even ridicule.^7^ What is more, in her recent annual report, the U.K. Forensic Science Regulator concluded that failing forensic science standards make “miscarriages of justice inevitable.”^8^

The intermediate interrogation at this point thus is how to move on from this impasse. In order to reflect further on this systemic relationship and assist in finding ways to cope with its difficulties, it is necessary to lay down some basic rules of conduct. Articulating the structural features of a normative platform favoring communication and mutual understanding between juridical decision-makers and expert witnesses can be seen as a mapping exercise, aiming at drawing normative borders and allocating epistemic rights and duties. Between fact-finders’ reaching beyond their legitimate scope of their expertise on the one hand and expert witnesses trespassing on the realm of the jury on the other hand, the challenge is to strike a scientifically defensible and jurisprudentially fair balance. But in order to avoid a mapping exercise to fall short of practical considerations, it is necessary to take into account the architecture of adversarial criminal adjudication,^9^ policy choices and methodological axioms of science. What is more, rethinking expert witness testimony has to take place during business-as-usual operation, avoiding interference with the established routines for generating, evaluating and validating knowledge claims in legal adjudication. Adjusting the normative structure of legal institutions to theoretically sophisticated models does not mean that one is authorized to change the structure of dispute resolution in autonomous legal orders. This perspective is not limited to criminal adjudication as forensic evidence can also play a crucial role in civil lawsuits (see, e.g., forensic document examination in the case *Zuckerberg v. Ceglia*).

### Law and Philosophy of Science

An analysis and discussion of forensic expert testimony is hardly possible without devoting some comments to the relationship between law and philosophy of science. The interest of the former in theories in general, and in questions such ‘what is science?’, is not restricted to academic circles. In fact, systems of legal adjudication are pragmatically – and in terms of substantive law – required to filter the admission of theories in their proceedings. This is a relevant observation because practitioners and forensic scientists will base propositions of interest, to some extent, on theoretical models. The principal issue with this is that in most jurisdictions it is the judge rather than the respective scientific community that will have to answer the question of (scientific) validity. Thus, introducing elements of philosophy of science at this juncture aims at further delimiting the focus of enquiry and clarify the nature and the scope of the decisional account introduced later in Section “Decisional Dimensions of Forensic Expert Testimony.”

The engagement of courts with philosophy of science has been hitherto rather light-hearted (Haack, 2014a, p. 141). But the academic discussion too, had difficulty to comprehend the scientific endeavor of expert witnesses against the background of the model-based view of scientific enterprise. The U.S. Supreme Court’s ruling on *Daubert*^10^ is particularly pertinent in this context as it has become a leading authority on monitoring the reliability and validity of expert evidence in all US Federal Courts and the majority of state jurisdictions. Most importantly, it has generated a remarkable amount of academic discussion on an international level, despite it not being directly relevant for proceedings in other jurisdictions. In *Daubert*, the petitioners (two minor children and their parents) had alleged that the children’s serious birth defects had been caused by a prescription drug marketed by the respondent. They also proposed to adduce evidence of the testimony of eight experts to the effect that the prescription drug can cause such side effects. Both the District Court and the Court of Appeals relied on Frye’s standard of admissibility,^11^ i.e., general acceptance, and declared the evidence inadmissible. For there was extensive published scientific literature on the subject that the maternal use of Bendectin has not been shown to be a risk factor for human birth defects.

The US Supreme Court declared that the rule of ‘general acceptance’ had been invalidated by the adoption of the Federal Rules of Evidence. The ruling signified departure from *Frye* and reliance on “general acceptability,” for the *Frye* test was superseded by the Rules’ adoption, in favor of a more liberal approach. According to Rule 703, Justice Blackmun who delivered the opinion of the Court said, the question that is most pertinent for the court is whether “all scientific testimony or evidence admitted is not only relevant, but reliable.”^12^ Let us stress that *Daubert* was about admissibility of the evidence, not weight. Undeniably, admissible evidence does not predetermine decisions. It changes, however, the dynamics of proof. The Court explicitly coupled evidentiary relevance qua precondition of admissibility and scientific validity. While the Court was at pains to stress that the central issue is validity, not a specific criterion thereof, it introduced a “flexible” inquiry encompassing multiple and non-exhaustive factors, which, the Court reminds us, do not “set out a definitive checklist or test.”^13^ Criteria such as whether the theory or technique underpinning the evidence has undergone testing and withstood the scientific process of falsifiability; whether it has been subjected to peer review and publication in refereed journals; whether there is information about its known or potential error rate; whether the theory or technique enjoys the support of some relevant scientific community or communities.^14^

Daubert has attracted wide criticism especially with regard to the Popperean criterion of falsifiability with authors going at great length to flesh out the philosophical argument. Allen (1994, p. 1164) concisely remarked that the Court “replaced a judicial anachronism [*Frye*] with a philosophical one [Popper].”^15^ Haack remarked, in the same tone, that Daubert’s “philosophy of science was confused” (Haack, 2014b, p. 113). To some extent, the unusual amount of criticism against *Daubert* overstates, in our opinion, the importance of a single member of a non-exhaustive set of criteria of validity, i.e., falsifiability. The inquiry enshrined in Rule 702, Justice Blackmun clarifies, is a flexible one and its overarching subject, to wit, scientific validity, cannot be reduced to any single criterion.^16^ Falsifiability, peer-review process etc. are simply indicators, not necessary and sufficient conditions of scientific validity. The major change that *Daubert* engineered is the shift from an externalist approach to scientific evidence to an internalist one. Whereas for 70 years judges would have to rely on the “general acceptance test,” they now need to comprehend the empirical claims and underlying methodology, for admissibility hinges on *asserted* scientific validity. It does not follow, thus, that the Court subscribed to Popper’s conception of science, for the Court did not answer authoritatively the question of what constitutes ‘validity.’ Secondly, *Daubert* is important for the contradistinction between science and legal adjudication. While “[s]cientific conclusions are subject to perpetual revision,” the Court points out, “law … must resolve disputes finally and quickly.”^17^ This remark raises more questions than it manages to answer. For one, it seems to assume that science is on a further end of some spectrum, investing more time and resources than legal adjudication does. If this is true and we can directly compare the two systems, one might wonder, then how are we to avoid the conclusion that scientific methods *are* superior to adjudicative ones? How could we justify the limited role expert witnesses are required to play, when modern legal orders equate parts of their testimony with trespassing on the province of the jury? To tackle these questions in the context of legitimacy of criminal adjudication, it is necessary to take a closer look at the philosophy of science encoded in *Daubert*.

Justice Blackmun seems thus to have placed some emphasis on the criterion of falsifiability, and has attracted waves of criticism ever since. A key question, he writes, in determining whether a theory or technique is scientific knowledge and therefore reliable and admissible in court, is whether it “can be falsified.”^18^ The Popperean scent ascending from the criterion of falsification is enhanced, for Justice Blackmun uses a direct quotation in his next sentence: “the criterion of the scientific status of a theory is its falsifiability, or refutability, or testability.”^19^ While Popper is a widely respected philosopher, especially among lawyers, his views have, however, never had an actual impact on existing and established methods for validation of scientific hypotheses. As Kuhn (1996, p. 77) remarked, “[n]o process yet disclosed by the historical study of scientific development at all resembles the methodological stereotype of falsification by direct comparison with nature.”

But the criterion of falsification was not the only hint to philosophy of science in *Daubert*. In the same paragraph Justice Blackmun mentioned also Carl Hempel, a central figure of Logical Positivism. This has been criticized as cherry-picking of philosophical ideas (Haack, 2014a). On the one hand, the criticism is justified, for there are major differences between Hempel’s verificationism and Popper’s falsificationism. On the other hand, the Supreme Court hit the nail on the head, apparently without even realizing it, because both meta-theoretical approaches share at least two structural features. Firstly, the presupposition that theories are based on some formal logical syntax, to wit, by carrying out axiomatization of theories within formal languages. Secondly, the idea that a scientific theory could be once and for all confirmed or falsified through a direct comparison with a theory-external criterion, i.e., a theory-neutral observational language. However, phenomenal appearances can only be validated in the light of a multitude of background assumptions (Jackson, 1988, p. 557). The theory ladenness of all experiential data, i.e., one of the radical insights of the second half of the 20th century, necessitated the abandonment of the strict divide between theoretical terms and observational ones. All in all, despite their differences, these two approaches, i.e., Hempel’s verificationism and Popper’s falsificationism, can be regarded from the stance of philosophy of science as the two main phases of the *syntactic view* of theories which dominated the first half of the previous century. The *syntactic view of theories* (Received View) with its phantasies of an ideal language comprising concepts with sharp boundaries, which dominated the field of philosophy until the 1950s, and its underlying logicism overestimated the power of formal logic. What is more, it failed to give a practicable account of actual and successful scientific theories (Suppe, 2000, p. S103). The latter are not axiomatic systems yielding deductively derived consequences, and scientific practitioners have more moderate requirements for scientific validity than a “relentless accumulation of confirming instances” (Toulmin, 1967, pp. 110–111). Scientific propositions are not based on strict unexceptional ‘laws,’ but on *generalizations*. The *semantic view* of theories, in turn, which has been dominant since the last quarter of the 20th century, is a formal reaction to the syntactic view of theories (Bailer-Jones, 2009, p. 126). It is remarkable, that both Courts and the academic discussion appear to have largely failed to register this major development, i.e., the fact that “[m]odels occupy central stage” (van Fraassen, 1980, p. 44) in philosophy of science.

Let us synthesize the above. According to the model-based view of scientific enterprise, theories are not empirically un-interpreted formal-axiomatic systems but involve a central interpretative aspect. In other words: theories are not fully axiomatized systems which eliminate the need for discretion in science let alone in legal adjudication. This highlights the need to outline the area of admissible interpretation for expert witnesses and fact-finders. Further, scientific models do not consist in the accumulation of instances who either confirm or fail to falsify a given hypothesis once and for all. An essential feature of modeling is to generalize. Generality, understood as the property of applying widely, plays a pivotal role in philosophy of science (Lewis and Belanger, 2015). Furthermore, it is important to understand the inversely proportional character of generality and precision. As Gleick (1998, p. 278) points out: “The choice is always the same. You can make your model more complex and more faithful to reality, or you can make it simpler and easier to handle.” This trade-off, however, is, no matter the outcome, subject to certain restrictions. The purpose of any model is to generalize and reduce reality to meaningful theoretical (i.e., general) propositions. A map which would be as accurate as the landscape itself would be a contradiction in terms. We can hold, therefore, that there is a point where any general account of the world breaks down. That point is the individual case. This insight, which is methodologically rather trivial but as regards its consequences radical, helps us realize the different dynamics and aspirations between legal adjudication and scientific endeavor.

## Decisional Dimensions of Forensic Expert Testimony

### Discretion in Law

#### Legal Conclusions and Decisions Versus Scientific Determinism: The Need for Discretion

As argued in Section “Transversal Overview of Current Empirical, Normative and Doctrinal Understandings of Expert Witness Testimony,” the function of any model providing scientific explanation is (i) to generate generalizable propositions (conclusions), presuming that “events occur in consistent patterns” (National Research Council [NAS], 2009, p. 111), (ii) to establish symmetry across members of a target system, and (iii) to eliminate the need for case-by-case treatment of individual cases. The validity of a general proposition, i.e., its scientific character, is a function of its derivability from a scientific model to such an extent that the very expression ‘*ad hoc* explanation’ strikes us as quite peculiar, indeed as a contradiction in terms. Singularities, where physical laws break down, are deeply troublesome for scientific theories. The question, then, is whether the fact-finder’s decision about unique historical events is also generalizable. From Aristotle, who observed that it is “foolish to accept probable reasoning from a mathematician and to demand from a rhetorician [i.e., lawyer] scientific proof,”^20^ to modern forensic scientists who are at pains to stress that the idea of a frequency being attached to an outcome for a single event is “ridiculous” (Lucy, 2006, p. 5), scholars have continuously rejected (bogus) claims of generality when it comes to legal decisions. However, we will need more than aphorisms, in order to draw a line between fact-finding in legal adjudication and scientific inquiry.

The fact that legal systems, especially in our increasingly complex world, are unable to, indeed not particularly interested in predicting and axiomatizing every combinatorial possibility of circumstances that the future may bring – this would be computationally intractable – is an enduring lesson we have learnt from the failures of legal orders that placed exclusive emphasis on casuistry and tried to provide an all-encompassing solution to the problem of decidability by enacting exhaustive lists of elements falling under a legal concept ‘*φ*’.^21^ The (vain) effort to provide an ontological map of semantics of legal concepts aimed at the elimination of discretion and predicated a fact-finder/judge who would effectively be ‘the mouth of the law.’ However, this presupposes a world comprising a finite number of features, so that we could lay down rules for each combination individually. As Hart put it, “[p]lainly this world is not our world” (Hart, 1961, p. 128). Explication of legal terms, including “proof,” is highly contextual. Securing a maximum degree of predictability (and therefore: legal certainty) comes at the price of “freezing” the meaning of legal terms by settling in advance issues before they arise. This would provide maximum legal certainty in lieu of paradoxical results, such as for example the prohibition of an ambulance entering a park, pursuant to the rule “*No* vehicles in the park.” The rigidity of various legal classifications, e.g. what constitutes ‘proof,’ would make legal orders instantly obsolete and unfit for resolving new questions that will inevitably emerge in litigation. In a world characterized by a radically unpredictable future, every deterministic approach to legal concepts – let us mention again that from the point of view of the law, ‘proof’ is a legal concept – would be in need of revision moments after its enactment in order to catch the multitude of situations that occur in real life, and keep abreast of social developments. The whole field of legal methodology and legal dogmatics grew out of the ashes of legislative projects that tried to eliminate discretion only to fail utterly.

Modern legal orders have internalized the message that it is futile to anticipate decisions.^22^ Admittedly, deduction from rules with predetermined meaning, elimination of discretion and the description of judge’s/fact-finder’s activity in logico-mechanical terms are features routinely attributed to juridical operations. The problem, however, is that these features derive from a rather superficial understanding of normative systems. Indeterminacy is not a surface feature of law but is inherent in natural languages. It is for this reason that logicians traditionally stress that truth values can be defined only within formal languages (Tarski, 1944). The dynamic process of increasing or decreasing the generality of legal rules inevitably runs into a point of bifurcation, where no decision either way is “dictated” by the applicable norm(s) (Hart, 1961). Notwithstanding the fact that the respective factfinder will probably have (good) reasons to reach the – in his or her opinion – right decision, from the point of view of the law there can only be a set of equally reasonable decisions. The applicable legal norm or standard in question is simply a “frame” within which various possibilities are given. The verdict, Kelsen explains, is on a thorough look an “individual norm,” valid exclusively with regard to the individual case, i.e., not generalizable (Kelsen, 1934, para 36). At this very point, axiomatized systems break down, for the fact-finder needs to make a decision, which is not warranted by the underlying logical framework. Particular cases, Hart remarks, do not make themselves fit for legal subsumption, “already marked off from each other,” or shouting at us: ‘I am an instance of the general rule’ (Hart, 1961, p. 126). Rules, including legal rules, do not provide the (meta-)rules for their own application.^23^ The gap between rational *conclusions* based on scientific models and personal *decisions* about disputed facts can only be filled by an *act of will* (Kelsen, 1934, para 5), which is not a necessary outcome of a justificatory chain. We will come back to this important point.

It is worth keeping in mind that an uncontradicted model-based proposition can be rejected only on pain of irrationality. This is the essence of the deference model in forensic science. A fact-finder cannot simply disregard the justified *conclusion* (decision structure) of an expert witness testimony, say, on the assigned probative value of some biological trace. However, decisions behave in a different way. They are not rationally resolvable, for reasonable minds may differ. Disagreement about the ‘one right decision’ does not necessarily imply an error in the justificatory process, since the logical chain of justification leads to a point of progress branching with mutually incompatible growing paths which the decision-maker can follow (Stegmüller, 1979, p. 33). The fact-finder has the epistemic duty to exercise discretion and resolve an issue by making a decision. Scholars who deny this fundamental insight are obliged to postulate caricatures of judges with “superhuman intellectual power” (Dworkin, 1986, p. 239). What is more, discretion is not an exclusive feature of law. The historian of science Kuhn has promoted a similar view. He emphasized the role of value judgments and decisions in the course of scientific development. E.g., debates over theory-choice, he says, “cannot be cast in a form that fully resembles logical or mathematical proof” (Kuhn, 1996, p. 199). There is no algorithm, e.g. for choosing the level of significance (3σ or 5σ), in an experiment.^24^ E.g., the existence of the Higgs particle is proved qua outcome of empirical research. Yet the underlying and staggering level of significance (5σ) is not itself a scientific fact; it is a convention and as such a matter of choice rather than of “purely theoretical reasons” (Stegmüller, 1979, p. 35).

#### The Values of (Criminal) Law

The previous considerations allow us to, first, articulate a principal source of confusion in discussions around expert witness testimony, and, secondly, explicate the decision-making prerogative alias *burden of decision*. Legal adjudication does not aim at, or aspire to answer empirical questions in a *general* way. It is not a shorter or less costly method of knowledge-claim validation. Its function and social task is to resolve, within a reasonable amount of time, a legal issue deriving from contested factual claims. Legal orders set general criteria, which, when met, authorize an official to impose a legal effect. The difficulty resides in the fact that the question *when* these general criteria (‘exclusion of reasonable doubts’ or ‘being sure’) are actually met, cannot be answered in the abstract. Social reality is complex and too context-sensitive for an algorithmic or axiomatized approach. Accordingly, the decision-making prerogative in actual cases refers to the responsibility to resolve an issue, not despite but because it is not replicable and therefore not subject to scientific analysis in the traditional sense. There is no univocal answer to the question of legal liability and proof because the question as such is not scientific, not because the underlying issue is obscure.^25^

Each legal order qua autonomous normative system will have to make a basic policy choice on who will take the responsibility and resolve a scientifically unresolvable – though scientifically describable – issue. The respective choice is not answerable to eternal and unalterable laws, but subject to historical contingencies, political balances and outcomes of social conflict. There is no *a priori* or scientifically valid reason to give the decision-making prerogative to professional judges, laypeople or experts, i.e., to opt for the educational or deference model. A decision is based on, albeit is not derivable by scientific propositions. It is pillared by the act of will of the respective official, who is authorized to make a decision, although no decision is logically necessitated by the underlying normative framework (Kelsen, 1934).

Utilizing an act of will does not mean that decision-making implies an anything-goes activity. Decisions are neither logically warranted, nor are they a step into the void. Each legal order has its own internal values, which the juridical decision-maker has to implement. The law especially criminal law and the criminal standard of proof are heavily influenced by policy considerations. The US Supreme Court has famously spelled out this dependency in the benchmark decision *In Re Winship*, which describes the reasonable doubt standard as “a prime instrument for reducing the risk of convictions resting on factual error.”^26^ The standard of proof (in a liberal legal order) reflects thus the increased social disutility of convicting a law-abiding citizen person. As Justice Harlan put it in his concurring opinion, the function of the standard of proof is to influence “the relative frequency of these two types of erroneous outcomes,” knowing that the two types of error (acquitting the perpetrator and convicting the innocent) are inversely proportionate.^27^ Similar considerations apply to almost every modern legal order.

Liberal legal orders, as opposed to authoritative ones, value the individualistic perspective, and the requirement that legal evidence has to be ‘specific’ cannot be sidestepped. As Justice Antonin Scalia put it, statistical evidence “is worlds away from [legally] ‘significant proof’.”^28^ The idea that some scientifically validated (general) proposition guarantees the factual and normative rectitude of a verdict (decision) creates a “major contradiction between the scientific status that is claimed and the operational paradigm to which its practitioners subscribe” (Champod and Evett, 2001, p. 101). It is worth reminding that related discussion exists in the area of clinical decision-making, where it has become increasingly clear that despite common assumptions, ‘diagnostic slam-dunks’ and absolute certainty are the rare exception rather than the rule. Given the inevitable element of uncertainty in a typical diagnosis, the physician will be able to express, in a warranted way, merely the probabilistic support for some medical condition, e.g. tuberculosis, as compared to relevant alternative hypotheses. But the evidence alone, and the subsequent grade of belief, will *not* necessitate what should be done (Sober, 2008, pp. 4–5). The primary interest of a patient is the choice of therapeutic measures, not the probability of any disease. As Sober remarks, answering the question ‘What should I do?’ requires more than data and grades of belief. It requires the input of values (Sober, 2008, p. 4). The question whether the diagnosed condition corresponds with the true state of affairs and the related question, which treatment should be preferred, requires and instigates an inferential leap. Prominent forensic scientists call this step “a leap of faith” (Stoney, 1991, p. 198). (Forensic) scientists are, therefore, not better equipped than laypeople, to take this step by making a decision under uncertainty.

### Forensic Reporting

#### Decision-Structures for Specific Evidence in Forensic Science

We can now exemplify our perspective on decision-structures by considering examples from forensic science reporting practice. We will focus on results of forensic DNA analyses that, despite critiques, are widely considered as a principal type of evidence, especially in criminal proceedings. The high variability of forensic DNA profiles between individuals has made it an attractive candidate for supporting claims of individualization. Traditionally, this goal has been conceived as the heart and soul of forensic science (Kirk, 1963, p. 236), and is also very common among other trace categories such as fingermarks, handwriting and the like, including also more recent trace types, such as digital traces.

Related to individualization is the notion of uniqueness which, however, is not an operable term in ordinary criminal adjudication. This hinges on methodological issues of the standard ways in which forensic scientists analyze biological traces. Forensic DNA profiling results reflect an individual’s genetic features at various points of comparison, the so-called loci. But since only a tiny part of the entire DNA-molecule is analyzed, an eventual correspondence between the profile of a crime stain and that of a person of interest is, per definition only partial, and does *not* establish that the person of interest is the source of the crime stain (Redmayne, 1995, p. 464). This is especially the case for incomplete DNA traces (e.g., degraded trace material), or mixtures of DNA composed of material from more than one contributor. The probative value of DNA profiling results will thus be explicitly *probabilistic*, and it is essential to understand that probative value is based on the notion of conditional genotype probabilities (hereinafter: CGP). The latter is a technical notion that expresses the probability of observing the DNA characteristics on the crime stain (i) given that an unknown person (i.e., different from the suspect or person of interest) is the source of the crime stain, (ii) given the task-relevant information available on the case file, and (iii) given additional considerations related to forensic genetic theory (Evett et al., 2000). The forensic biologist’s assessment thus focuses on the probability of observing corresponding DNA characteristics in an unknown person from the relevant population (i.e., the population to which the source of the crime stain is thought to belong), which may be Caucasians, Chinese etc.^29^ The probabilistic character of the respective report highlights the importance of distinguishing sharply between using a model to describe, *in a general way*, a phenomenon (i.e., the kind of genetic features observable on the crime stain) on the one hand, and using such information in order to make a legally structured and procedurally contextualized *decision* on the other hand. This categorical distinction has already been drawn by criminal courts.^30^ Schematically, thus, two different questions regarding DNA evidence are commonly of interest:

(1) What is the probability that an individual will be observed to have the DNA profile features of interest as seen in the trace *given that this person was chosen at random from the population of interest*?
(2) What is the probability that a given individual is truly the offender (or, the source of the crime stain), *given that corresponding DNA features between the profile of that person and the profile of the crime stain have been reported*?

It is worth mentioning that in the *first* question, the factual innocence of the person of interest is taken for granted and one asks what the CGP is, whereas in the *second* question one takes the corresponding DNA features (i.e., the forensic findings) for granted and envisages the ascription of criminal liability (or, inference of source). As anticipated in Section “Key Controversies Over Selected Aspects of Forensic Expert Testimony,” the widely known prosecutor’s fallacy consists in transforming the answer to the first question into an answer of the second one. The prosecutor’s fallacy is, however, not the only misinterpretation that one may make in relation to the above two questions. The further source of methodological error (and procedural violation) is what may be referred to as the ‘expert witness’s fallacy,’ which refers to the situation in which forensic experts testify even further beyond the area of their expertise. This occurs, specifically, when experts purport to answer *both* questions, although they are legitimized to answer only the first. This sidesteps the understanding that “a sharp distinction can be made between what one ought to think about a proposition […] and what one actually decides […] the former is a problem that pertains to probabilistic reasoning whereas the latter is one that applies to decision making” (Biedermann et al., 2008, p. 23).

Interestingly, though not coincidentally, the above two separate questions map on the distinctive epistemic duties for expert witnesses and jury. The expert witness, the Court of Appeal in England and Wales makes clear, “should not be asked his opinion on the likelihood that it was the defendant who left the crime stain, nor when giving evidence should he use terminology which may lead the jury to believe that he is expressing such an opinion.”^31^ The expert witness, in other words, is logically warranted and legally authorized to express only the information regarding the probative value of the scientific findings (*not* an opinion regarding the truth or otherwise of the propositions of interest). Such information may take the form of, for example, the CGP, or a measure that is a function thereof. The situation is different, however, with the second question. Even in the factually remote, but epistemically possible case, where the correspondence would extend to more features than are included in traditional DNA profiles, and the source of the DNA is not contested by the adversarial parties, the question of liability would still not have been answered. Further considerations need to be taken into account for reasoning to such higher propositional levels, such as the relevance of the evidential material for the offense of interest (Stoney, 1994). In essence, associating a person of interest with evidential material, as such, does *not* answer any ultimate issue (i.e., a substantive element of an offense).^32^

This does not necessarily mean that the expert witness would be excluded in advance from answering non-scientific questions. The point merely is that there is no obvious reason to believe that expert witnesses would be any better than laypeople in answering questions such as individualization or liability. As Cole puts it, “the expert has no special competence greater than that of any other person at the decision stage of the process” (Cole, 2014, p. 143). Practically, one should even expect experts to be in a *less* informed position compared to the jury, because only the latter oversees the case as a whole. But again, it is the divide between reasoning about propositions, on the one hand, and actually making a decision regarding those propositions of interest, on the other hand, which poses both conceptual and procedural hurdles, and this clarifies why the expert is not in the position to act at the stage of decision. Guilt is not a (scientific) proposition, but a verdict, which is the result of a decision under uncertainty made after considering all elements of the case. The allocation of the decision-making prerogative is, intrinsically, a policy choice rather than a scientific mandate. Legal orders may choose freely whom they entrust with this important legal duty of deciding on the defendant’s liability, without violating any logical or methodological principles of scientific inquiry.

Forensic scientists who – on an industrial scale – make categorical claims in terms of so-called individualization conclusions (see Section “Key Controversies Over Selected Aspects of Forensic Expert Testimony”) with respect to the defendant, to the exclusion of all others, are wrong both in terms of methodological underpinnings and the law. They seem to conflate individualization qua ontological claim – according to which there is no other person who could be found to correspond to the *particular* DNA profile observed in the case at hand – and individualization qua epistemic claim leading to a definitive conclusion that has potentially decisive impact on the verdict (Saks and Koehler, 2008, pp. 211–219).^33^ It is a longstanding, though still not widely appreciated, fundamental insight that for moving from the evidence to an individual as the proclaimed source of an item or trace, a leap of faith is required. To this we add, through our discourse here, that such forensic conclusions can also be framed as decisions, requiring an act of will (see Section “Decision-Structures for Specific Evidence in Forensic Science”). This adds further support to the argument that requiring expert witnesses to confine themselves to their area of responsibility as outlined from the respective legal order is not a deliberate dogmatic choice, but both a logical and procedural necessity. Anything else would amount to trespassing onto the province of the jury.^34^

By arguing that the concept of individualization, salient in forensic science, actually comes down to a decision, and as such hinges on an act of will, it is not denied that a decision can and should be scientifically backed. The point solely is that the scientific model used to articulate the respective target system is only a *conditio sine qua non* for any decision in a system of legal adjudication with commitments to Rationalism. It is not, however, a *conditio per quam* for the respective decision. Expert witnesses inform and educate the fact-finder/juridical decision maker but are not entitled to anticipate their decision. As Lord President Cooper put it, it is the expert witness’s duty “to furnish the judge or jury with the necessary scientific criteria for testing the accuracy of their conclusions, so as to enable the judge or jury to form their own independent judgment by the application of these criteria to the facts proved in evidence.”^35^ This is a matter of actual and contingent and yet valid policy choice, not a misguided and sub-rational pre-scientific operation. Interestingly, even the pioneering forensic scientist Locard supported the view that the laboratory should not become the “antechamber” of the court (Locard, 1940).

Notwithstanding, there is an intrinsic connection between reasoning based on incomplete items of evidence on the one hand, and acts of will on the other hand, leading to decisions: the two instances are connected in the sense that the former is the point of departure of the latter (Biedermann et al., 2008). As much as the scientist cannot interfere with the judicial decision-makers’ area of competence, the juridical decision-maker cannot interfere with the process leading to the expert witness testimony, especially its content. The focus, Justice Blackmun remarks, “must be solely on principles and methodology, not on the conclusions that they generate.”^36^ As long as these decision-structures are a function of valid scientific methods, they cannot *per se* be rejected or disregarded. Scientific conclusions cannot, however, anticipate ultimate issues, i.e., elements of the respective offense to be proved, let alone the verdict as such (Roberts and Zuckerman, 2010, p. 490).

#### The Role of Formal Theories for Reasoning and Decision Analysis

Through Sections “Transversal Overview of Current Empirical, Normative and Doctrinal Understandings of Expert Witness Testimony” and “Decisional Dimensions of Forensic Expert Testimony” we have aimed at clarifying the intricacies of the systemic relationship between forensic science and the law. Naturally, this raises questions from a variety of viewpoints – normative, descriptive and prescriptive – that, in many discourses on the topic, are not well separated, and hence hinder progress toward a resolution of opposing views.

Many of current debates focus on empirical and descriptive aspects, such as the question of the extent to which witnesses are testifying on the basis of knowledge, and whether the fact-finders can appropriately assess such testimony to reach sound judgments about the disputed events. It is, however, equally important – in our view – to insist on the understanding that structural features of expert witness testimony are actually embedded in a legally structured decision-making process. There are currently two main perspectives in which the decisional dimension of expert witness testimony may be understood.

On an empirical account, claims have been raised that forensic scientists should be subjected to empirical testing. As noted by PCAST, “studies are required, in which many examiners render decisions about many independent tests (typically, involving “questioned” samples and one or more “known” samples) and the error rates are determined” (President’s Council of Advisors on Science and Technology [PCAST], 2016, p. 143). Such research leads to general measures of expert performance with respect to a particular area of expertise and/or a given expert’s performance. The benefit of this is the provision of information to help assess whether experts, including their methods and techniques, are able to do what they claim to do, and whether they are any better in their tasks than lay persons. The obvious limitation of the empirical perspective is, however, that such general expert performance measures do *not* instruct, normatively, how to make a sound decision (i.e., what to conclude at the end of a forensic examination) in any given individual situation. The latter has to do with the logic of decision and, hence, requires elements of formal methods of reasoning and analysis, among which a prime candidate is (normative) decision theory. Broadly speaking, the purpose of decision theory is to assist decision-makers – in any given case – in thinking about the relative merit of rival courses of action when their outcomes cannot be known with certainty. While this appears to be a good fit for the needs of operational decision makers, there is debate over the possible uses of decision theory since its first presentation in legal literature in the late 1960s. A recurrent critique is that one of the entailments of decision theory, such as expected utility/loss, is not a relevant criterion from a legal point of view because that there are several other aspirations to which the legal process seeks to conform. It is important to note, however, that this is a critique that focuses on the descriptive and prescriptive adequacy of decision theory, while leaving the fundamental question of how to actually make a decision, and justifying it, unresolved. As the eminent figure in decision theory, Howard Raiffa, has noted: “Even if you don’t analyze your decision problem by the methodology described in these lectures, you still must act. What will you do?” (Redmayne, 1995, p. 272). We also see here a practical instance of the generality tradeoff mentioned earlier in Section “Law and Philosophy of Science”: formal theories assert coherence *at the level of detail* at which they are applied, which depends on the decision analyst’s intentions, (computational) capacities and available resources (e.g., in terms of information, time, etc.). It is pointless then to either claim a particular modeling result as a solution for a larger decision problem, or in turn criticize the model for a lack of completeness that it never claimed to have.

We join the above perspective in the sense that it places the inevitability of decision in the first place, and as the overarching perspective (see also Lindley, 1985). This burden of decision, as we call it, has to be absorbed, from which follows the imperative that decision-makers ought to think about their decision problems sensibly, prior to making their decision. The role of formal theories in this task is that of helping individuals make up their minds, in a structured way, about the fundamental ingredients of decision problems (i.e., states of nature, decisions, consequences, etc.). There is nothing prescriptive in this perspective as such, though it provides us with a critical analytical account of current practice. To illustrate this, reconsider one of the currently most controversial forensic reporting issues, that is the problem of *deciding* whether or not a defendant, rather than an unknown person, is the source of, for example, a DNA trace, a partial fingermark or an item of handwriting – a process commonly known as individualization (see Section “Key Controversies Over Selected Aspects of Forensic Expert Testimony”). Our general argument throughout this paper, emphasizing the judiciary’s decision-making prerogative, and the imperative to consider all findings, not only scientific findings, is to deny scientists answering this question. Firstly, because it would be an answer provided on an issue (i.e., a proposition of interest), rather than a statement of the value of forensic findings only. Secondly, because deferring the decision to scientists would lead them into an impasse. The impasse is due to the fact that any decision taken in the light of uncertainty is bound to decision consequences, some of which are undesirable (e.g. a false identification), and there is nothing in the scientists’ scope of competence that entitles them to assess the relative desirability or undesirability of those consequences (Biedermann et al., 2008, 2016), neither qualitatively and even less so quantitatively. The problematic turn on this is that scientists who continue to make identification decisions, despite this intricacy, will implicitly impose a stance with respect to possible decision consequences to the judiciary, without telling them that they do so, which raises problems of transparency. An even further dimension of concern is that scientists may not even be aware of the decisional dimensions, and their implications, of their form of testimony. Taken together, these intricacies have been recognized as the principal reason why forensic ‘identification practice’ has become unscientific (Stoney, 1991, 2012).

## Discussion and Conclusion

In their paper on the “Individualization Fallacy” Saks and Koehler (2008, p. 215) wonder why so many forensic scientists “ascribe greater powers to their fields than the research supports.” A Nietzschean “will to power,” masqueraded as individualization claims, lack of understanding^37^ for the structure of legal adjudication, the probabilistic (general) character of scientific propositions, or simply an aspiration to ‘help solve the crime’ are only a few possible answers. Our paper does not aspire to answer this (empirical) question. Further, it is not helpful to fall back in disputes between mainstream evidence scholarship and forensic scientists. Efficient synergy between decision-makers and expert witnesses is too crucial for any modern criminal justice system to be conceptually or even institutionally crippled by a lack of communication and mutual understanding of respective roles and duties of the participants in the legal process. We purported, thus, to clarify – descriptively and analytically – the dimensions in which scientific knowledge, data and related expert assessments manifest themselves in different operative systems, i.e., legal adjudication on the one hand and core scientific theory and practice on the other hand. The conceptual boundary between model-based scientific conjectures and legally contextualized decisions outlines, at the same time, the allocation of epistemic duties and rights between expert witnesses and decision-makers (fact-finders). This perspective diverges from and goes beyond traditional discourses reduced mainly to questions such as admissibility and weight of particular items of scientific evidence because even if the latter issues are settled, the fundamental question on how scientific evidence interfaces with decision making in operational contexts remains an unresolved applied problem. Stated otherwise, even if agreement can be found as to whether an expert witness is appropriately testifying on the basis of knowledge, the fact-finder will still need to intelligently incorporate the witness’s testimony in the process of reaching a judgment about the contested events.

When conclusions of forensic scientists do not confine themselves to the scientific findings and their assigned probative value, but amount to categorical assertions about propositions (i.e., ‘this person is the source of this crime stain’), and hence represent *local* decisions (to be distinguished from *ultimate* decisions), the precept of factfinders controlling the decision process is violated. As much as ultimate inferential conclusions (e.g., about the defendant’s liability) are never based solely on the probability of particular propositions of interest, but also involve aspects of juridical classification (Roberts and Zuckerman, 2010, pp. 133–137), conclusions about lower propositional levels (e.g., inference of source; see Section “Key Controversies Over Selected Aspects of Forensic Expert Testimony”) involve value judgements regarding the risk of decision consequences (e.g., false identification and, hence, false incrimination of a defendant).

While not prescribing an answer to the above issues, the role of formal methods of reasoning and decision analysis – such as decision theory (Biedermann et al., 2008, 2016) – is to bring these underlying tenets to the open, and to clarify what is fundamentally at stake with any forensic conclusion. The insight from such formal analysis shows, in particular, that the scope and implications of forensic conclusions are much broader than what is commonly thought, because of the required value judgements (e.g., in terms of utilities/losses). The latter call upon a more active role by participants of the legal process other than the scientist. The understanding of forensic expert conclusions in a decisional dimension thus can empower the different parties in the process by showing that the processing of scientific evidence is a task that encompasses broader considerations than those that a scientist alone may address. This is entirely compatible with the view that the ultimate assessment of “any particular piece of evidence, scientific or otherwise, must always be assessed contextually, in the light of its contribution to the case as a whole” (Aitken et al., 2010, p. 70).

It remains the question of what role and position research in judgment and decision making may have in the context the legal adjudication. Over the past decades, literature on this topic has developed extensively and in great depth, and with proponents arguing in controversy about the merits of theoretical research when considering the dynamics of real trials and the limitations of what participants in the legal process are actually capable of doing. These discussions led to valid points to be made from all common analytical viewpoints, normative, descriptive and prescriptive. In this paper, we have extended this perspective to the particular interface between forensic science and the law where, traditionally, the form and content of expert conclusions have attracted critical discussions mainly in a probabilistic perspective, but without giving due consideration to the fact that expert testimony actually amounts to decisions being made with respect to target propositions (e.g., concluding that ‘this trace comes from this person’). Rethinking traditional forensic reporting practices, in particular source identifications, in this decisional dimension leads to two main conclusions.

First, while decision-makers in the context of legal adjudication need a scientific basis as a starting point, scientific models and forensic practitioners can at best facilitate the cognitive access to empirical phenomena by providing a systematic account going beyond common knowledge and understanding, i.e., a decision-structure. However, decision-makers need to “jump” (Stoney, 1991, p. 198) in order to render a verdict. As much as model-based propositions (scientific *conclusions*) cannot preempt *decisions* such as the ascription of (criminal) liability, they also cannot preempt decisions regarding forensic source attribution (i.e., concluding that a particular trace or mark comes from a designated person of interest). The main reason for this is that such conclusions depend on more than scientific or other evidence alone. Moreover, modern legal orders choose unequivocally and consistently to allocate the decision-making prerogative to fact-finders (professional judges or jurors), with a clear preference to education over deference.

Second, analyzing expert witness testimony through the lenses of formal theories, in particular normative decision theory, shows that the above allocation of duties and prerogatives actually makes sense; however, the analysis does not claim to practically facilitate the operation of the expert and fact-finder interface. The latter is not a drawback of judgment and decision-making research, but an insight that is valuable to guide ongoing reforms of forensic science reporting practice (expert witness testimony), as evidenced by the recent examples of scholarly works and policy documents drawn from professional bodies and governmental institutions presented throughout this paper.

## Table of Cases

*Davie v. Edinburgh Magistrates* [1953] SC 34, 40.

*Daubert v. Merrell Dow Pharmaceuticals, Inc*., 509 U.S. 579 (1993).

*Frye v. United States*, 293 F. 1013 (D.C. Cir. 1923).

*In re Winship*, 397 U.S. 358 (1970).

*R. v. Deen*, The Times January 10.

*R v Doheny and Adams* [1997] 1 Cr App R 369, 374.

*R v Gilfoyle* [2001] 2 Cr App R 57, 67, CA.

*R v Ireland; R v Burstow* [1997] 3 WLR 534.

*Wal-Mart Stores, Inc. v. Dukes, et al*., 564 U.S. 338 (2011).

*Zuckerberg v. Ceglia*, U.S. COURT OF APPEALS, 2nd cir., Apr 20, 2015, No. 14-1365-cv.

## Author’s Note

This paper has been presented at the 3rd International Symposium on Sino-Swiss Evidence Science (‘Pursuing Truth from Different Perspectives’) in Hangzhou (China), organized by the China University of Political Science and Law (Beijing, China), Guanghua Law School (Zhejiang University, China) and the School of Criminal Justice (University of Lausanne, Switzerland).

## Author Contributions

Both authors listed have made equal, substantial, direct and intellectual contributions to the work, and approved it for publication.

## Conflict of Interest Statement

The authors declare that the research was conducted in the absence of any commercial or financial relationships that could be construed as a potential conflict of interest.

## Footnotes

1 In this paper, we understand the term ‘normative’ not in a legal sense (i.e., referring to a legal norm or precept), but in the way commonly understood in the JDM literature of applied psychology, that is as a logical standard against which people’s reasoning and decision making can be evaluated (e.g., Baron, 2008, 2012). See also Hahn (2014) and Oaksford (2014) for accounts of normativism in the JDM literature.

2 By decision outcome we mean a decision taken in the light of a particular state of nature. For example, the decision to convict a truly liable defendant is an accurate decision outcome, whereas a guilty verdict for a truly innocent defendant is an inaccurate decision outcome (i.e., a false conviction).

3 See, e.g., Allen (2015) for an account on dimensions such as the organization of trials, governance, social concerns, and enforcement issues.

4 Note that similar documents exist in other jurisdictions. An example is the German ‘Standard of fingerprint identification’ [translation by the authors; original title: ‘Standard des daktyloskopischen Identitätsnachweises’] [BKA (Bundeskriminalamt), 2010].

5 For a timely and critical review, see Cole (2018).

6 *R v Ireland; R v Burstow* [1997] 3 WLR 534, House of Lords, per Lord Steyn.

7 See e.g. the recent negative publicity blitz strike at Jon Oliver’s political satire show on the shortcomings of forensic science, Last Sunday Tonight, Season 4, Episode 25 (2017).

8 See https://www.theguardian.com/uk-news/2018/jan/19/uk-police-forces-failing-to-meet-forensic-standards-safe-regulator-miscarriages-justice-outsourcing (01.25.2018).

9 We do not wish to make any specific claim on the differences between adversarial and continental systems of adjudication, given that from our structural point of view, differences tend to disappear. For reasons of simplicity and in order to provide concrete and comprehensible examples we will focus on adversarial systems of criminal adjudication. See Damaska (1973) for more discussion.

10 *Daubert v. Merrell Dow Pharmaceuticals*, 509 U.S. 579 (1993).

11 *Frye v. United States*, 293 F. 1013 (D.C. Cir. 1923). According to the old Frye Rule, novel scientific testimony needed to be generally accepted in the relevant field in order to be declared admissible.

12 *Daubert v. Merrell Dow Pharmaceuticals*, 509 U.S. 579 (1993), at 589.

13 *Id*., at 593.

14 *Id*., at 580.

15 Allen is in turn citing one of his students.

16 *Daubert v. Merrell Dow Pharmaceuticals*, 509 U.S. 579 (1993), at 594.

17 *Id*., at 597.

18 Id., at 593.

19 Id., at 593; Popper (1962, p. 37).

20 Aristotle Nic Ethics i3, 1094b.

21 The fact that legal systems operating in complex environments are unable to anticipate the future and contain rules allowing for exceptions incapable of exhaustive statements is a historic lesson we have learnt at least since the Prussian Legal Code (1794) with its more than 19,000 paragraphs.

22 For example, the Criminal Law Revision Committee for England and Wales [CLRC] (1980) [Fourteenth Report, Offences against the Person (1980), Cmnd 7844, para 37] emphasized that they are “extremely hesitant about embodying in a statute (which is not always susceptible of speedy amendment) an expression of present medical opinion and knowledge derived from a field of science which is continually progressing and inevitably altering its opinions in the light of new information.”

23 Interestingly, the same holds for formal analytical frameworks, such as probability and decision theory.

24 However, there is argument, at least in forensic science, to the effect that frequentist significance levels ought *not* to be used (e.g., Taroni et al., 2016).

25 For a clarification of this point in the context of forensic identification see, for example, Biedermann et al. (2008, Section 5.2).

26 *In re Winship*, 397 U.S. 358 (1970), Opinion of the Court (Brennan, J.), at 363.

27 *Id*., Harlan, J., Concurring Opinion, at 371–372.

28 *Wal-Mart Stores, Inc. v. Dukes, et al*., 564 U.S. 338 (2011), Opinion of the Court (Scalia), at 14.

29 Such probabilities may become increasingly small, in particular smaller than one over several world populations, intriguing some commentators, including scientists, to assert individualization (i.e., uniquely assigning a person as the source of a given biological trace). However, such claims are unfounded because they take values produced by a biological *model*, operated at an extreme end of extrapolation and, thus, beyond what may be empirically investigated, as face values.

30 *R. v. Deen*, The Times January 10.

31 *R v Doheny and Adams* [1997] 1 Cr App R 369, 374.

32 Rule 704(b) makes this point explicit when it states that “[i]n a criminal case, an expert witness must not state an opinion about whether the defendant did or did not have a mental state or condition that constitutes an element of the crime charged or of a defense. Those matters are for the trier of fact alone.”

33 The (National Research Council [NAS], 2009, p. 43) remarks on a similar note that the “question is less a matter of whether each person’s fingerprints are permanent and unique – uniqueness is commonly assumed – and more a matter whether one can determine with adequate reliability that the finger that left an imperfect impression at a crime scene is the same finger that left an impression … in a file of fingerprints.”

34 In view of that distinction Swinton Thomas *LJ* (*R v Davies*) remarked that it is “fundamental that experts must not usurp the functions of the jury in a criminal trial.”

35 *Davie v Edinburgh Magistrates* [1953] SC 34, 40; nota bene this is a Scottish case, which became also the leading authority in England and Wales, see e.g. *R v Gilfoyle* [2001] 2 Cr App R 57, 67, CA.

36 *Daubert v. Merrell Dow Pharmaceuticals, Inc*., 509 U.S. 579 (1993), Syllabus, at 580.

37 Margot (2011, p. 796) has concisely expressed this as follows: “Forensic scientists are proud to see themselves take such an important part in legal proceedings, failing to recognize that they’re playing the tune of their masters.”
